# Impact of the Body Composition on Knee Osteoarthritis Assessed Using Bioimpedance Analysis

**DOI:** 10.3390/jcm12227037

**Published:** 2023-11-10

**Authors:** Jaromir Jarecki, Bartosz Potoczniak, Artur Dziedzic, Teresa Małecka-Masalska, Tomasz Skrzypek, Waldemar Kazimierczak, Marcin Skowronek, Magdalena Wójciak, Sławomir Dresler, Marcin Waśko, Ireneusz Sowa

**Affiliations:** 1 Department of Rehabilitation and Orthopaedics, Medical University of Lublin, 20-059 Lublin, Poland; 2 Department of Orthopaedics and Traumatology, Regional Hospital in Chełm, 22-100 Chełm, Poland; b.potoczniak@gmail.com; 3 Department of Orthopaedics and Traumatology, Regional Hospital in Tarnobrzeg, 39-400 Tarnobrzeg, Poland; arturdanieldziedzic@gmail.com; 4 Physiology Department, Medical University of Lublin, 20-059 Lublin, Poland; tmalecka@gmail.com; 5 Department of Biomedicine and Environmental Research, Faculty of Medicine, John Paul II Catholic University of Lublin, 20-708 Lublin, Poland; tomasz.skrzypek@kul.pl (T.S.); waldemar.kazimierczak@kul.pl (W.K.); marcin.skowronek@kul.pl (M.S.); 6 Department of Analytical Chemistry, Medical University of Lublin, 20-059 Lublin, Poland; magdalena.wojciak@umlub.pl (M.W.); slawomir.dresler@umlub.pl (S.D.); ireneusz.sowa@umlub.pl (I.S.); 7 Department of Plant Physiology and Biophysics, Institute of Biological Sciences, Maria Curie-Skłodowska University, 20-033 Lublin, Poland; 8 Department of Radiology and Imaging, The Medical Centre of Postgraduate Education, 01-813 Warsaw, Poland; marcin@wasko.md

**Keywords:** osteoarthritis, knee, postmenopausal females, bioimpedance analysis

## Abstract

Osteoarthritis (OA) ranks among the most prevalent inflammatory diseases affecting the musculoskeletal system and is a leading cause of disability globally, impacting approximately 250 million individuals. This study aimed to assess the relationship between the severity of knee osteoarthritis (KOA) and body composition in postmenopausal women using bioimpedance analysis (BIA). The study included 58 postmenopausal females who were candidates for total knee arthroplasty. The control group consisted of 25 postmenopausal individuals with no degenerative knee joint changes. The anthropometric analysis encompassed the body mass index (BMI), mid-arm and mid-thigh circumferences (MAC and MTC), and triceps skinfold thickness (TSF). Functional performance was evaluated using the 30 s sit-to-stand test. During the BIA test, electrical parameters such as membrane potential, electrical resistance, capacitive reactance, impedance, and phase angle were measured. Additionally, body composition parameters, including Total Body Water (TBW), Extracellular Water (ECW), Intracellular Water (ICW), Body Cellular Mass (BCM), Extracellular Mass (ECM), Fat-Free Mass (FFM), and Fat Mass (FM), were examined. The study did not find any statistically significant differences in the electrical parameters between the control (0–1 grade on the K–L scale) and study groups (3–4 grade on the K–L scale). However, statistically significant differences were observed in BMI, fat mass (FM), arm circumference, triceps skinfold thickness, and sit-to-stand test results between the analyzed groups. In conclusion, the association between overweight and obesity with KOA in postmenopausal women appears to be primarily related to the level of adipose tissue and its metabolic activity.

## 1. Introduction

Osteoarthritis (OA) is one of the most prevalent inflammatory diseases of the osteoarticular system and one of the leading causes of disability worldwide, affecting approximately 250 million people [1]. The knee joint is on the list of human joints that are highly prone to degeneration due to its location, function, and proneness to injuries [2,3]. Osteoarthritis (OA) of the knee is a progressive disorder of the knee joint caused by the gradual loss of cartilage and resulting in the development of bony spurs and cysts at the joint margins [4]. The etiology of OA is multifactorial and involves inflammatory, metabolic, and mechanical factors [5]. The two main risk factors for knee OA are female gender and obesity. The incidence of OA has a predilection to the female gender with a ratio of about 1.7:1 [6].

In postmenopausal females, some changes in body composition are not necessarily associated with an increase in body weight but with a disturbance in the fat-to-lean body mass ratio [7].

Although there is a clear link between obesity and knee osteoarthritis severity, the exact mechanism of this relationship has not been elucidated to date. Increased body weight exerts an evident effect on the progression of degenerative lesions through biomechanical action; however, degenerative changes are also detected in unloaded joints, e.g., small hand joints [8].

Obesity is connected with low-grade inflammation, and the loss of cartilage may be caused by an imbalance between pro- and anti-inflammatory cytokines, resulting in catabolic effects. Adipose tissue is related to OA development and progression via biomechanical, metabolic, and pro-inflammatory factors triggering knee OA. It has emerged as a potent internal endocrine organ based on its ability to secrete biologically active adipokines, such as leptin and adiponectin, which are involved in different physiological processes [2].

The body mass index (BMI) is used for the assessment of overweight and obesity. In turn, bioimpedance analysis (BIA) is used for detailed analyses of the distribution of adipose tissue and its dependence on other body tissues. This non-invasive, inexpensive, quick, and reproducible examination is not burdensome to the patient [9]. BIA can be used to measure many body composition parameters. It is based on the assumption that electrical current is well conducted by tissues containing large amounts of water and electrolytes but poorly conducted by adipose tissue and bone mass [10].

The aim of the present study was to assess potential relationships between the severity of knee osteoarthritis and the body composition (fat and lean tissue) measured via BIA in postmenopausal females.

## 2. Materials and Methods

### 2.1. Selection of Patients

The study was carried out at the Department of Orthopedics and Traumatology in Tarnobrzeg in 2017–2018 and at the Trauma and Orthopedic Department of the Provincial Specialist Hospital in Chełm in 2022–2023. The study was approved by the Bioethics Committee (Resolution No. KE-0254/48/2014 of 27 February 2014). Written informed consent was provided by all participants.

The study involved 58 postmenopausal females qualified for total knee arthroplasty due to degenerative joint changes. The inclusion criterion for the study group was the presence of degenerative changes with a degree of 3 to 4, as assessed based on radiological images. The control group comprised 25 postmenopausal patients with no degenerative changes in the knee joints confirmed by radiological images (degree of 0 to 1).

X-ray images in two projections of the standing position, anterior–posterior (A-P) and lateral, were taken in all patients. The degree of degenerative changes in both groups was assessed by two surgeons with the use of the Kellgren–Lawrence scale [11]. The Kellgren and Lawrence system is a common method of classifying the severity of KOA using five grades. Its original description is as follows (Table 1):

The exclusion criteria were as follows: male gender, infection within 3 months prior to the study, active deep vein thrombosis in the lower limbs, active dental and periodontal diseases, skin lesions at the electrode application sites, past amputation, limb edema due to renal or cardiovascular causes, pacemaker implantation, and failure in post-implantation of prostheses or orthopedic implants. In addition, pre-menopausal women under 49 years of age, women with menopause occurring less than a year before the study, patients with rheumatological, endocrinological, and neurological comorbidities, patients receiving estrogens, glucocorticoids, or thyroid hormones, and individuals who were dieting or exercising were also excluded.

### 2.2. The Anthropometric Assessment

The anthropometric analysis assessed the body mass index (BMI (kg/m^2^) = b.w. (kg): (height in m)^2^) as well as mid-arm and mid-thigh circumferences (MAC and MTC). Additionally, the thickness of the triceps skin fold (TSF) was measured by pinching the skin and underlying tissues above the triceps of both upper extremities with a skin fold gauge. A Slim Guide Skinfold Caliper (Creative Health products, Ann Arbor, MI, USA) was used for the skin fold measurements. The triceps measurement was taken at the mid-point between the acromial process and the radial prominence of the radius, with the arm relaxed and positioned at the side of the body with the hand supine. Skin and AT were pinched between the thumb and forefinger in a vertical manner, ensuring that only skin and AT were contained. The caliper was applied at 90 degrees to the pinch, 1 cm from the fold edge, and the measurement was recorded in millimeters (mm).

### 2.3. Sit-to-Stand Test

The functional performance of the patients was assessed using the 30 s sit-to-stand test. It was conducted using a standard (46 cm) height chair, with the subject seated in the middle of the chair and her feet on the ground. The participant was instructed to stand from a seated position as many times as possible in 30 s; the total number of full repetitions was recorded. One full repetition began and ended with the participant seated [12].

### 2.4. Electrical Bioimpedance Assessment

**Figure 1:**
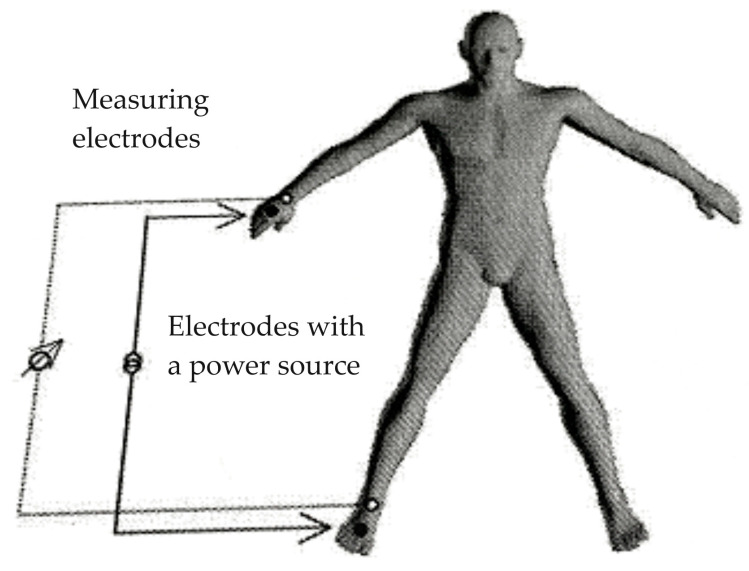
Standard electrode application sites on the hand, wrist, foot, and ankle in the tetrapolar system.

Electrical bioimpedance analysis (BIA) was performed using the ImpediMed bioimpedance analysis SFB7 BioImp v 1.55 device (ImpediMed, Carlsbad, CA, USA) in accordance with the manufacturer’s instructions. Standard electrode application sites are shown in Figure 1 [13]. During the BIA test, the following electrical parameters were assessed: membrane potential (C membrane), electrical resistance (R—resistance) parameters measured at current frequencies of 5, 50, 100, and 200 kHz, capacitive reactance (reactance—Xc), impedance (Z), and phase angle—(Ph—Φ), and body composition parameters: Total Body Water (TBW), Extracellular Water (ECW), Intracellular Water (ICW), Body Cellular Mass (BCM—Body Cell Mass), Extracellular Mass (ECM), Fat-Free Mass (FFM), and Fat Mass (FM).

### 2.5. Statistical Analysis

Statistical analysis was performed using GraphPad Prism 8.4.3 (GraphPad Software, Inc., San Diego, CA, USA). The normality of data distribution was evaluated using the Shapiro–Wilk test. The *t*-test was used to analyze normally distributed variables, while non-normally distributed data were analyzed using the Mann–Whitney U test.

## 3. Results

### 3.1. Characteristics of the Patients and Functional Tests

**Figure 2:**
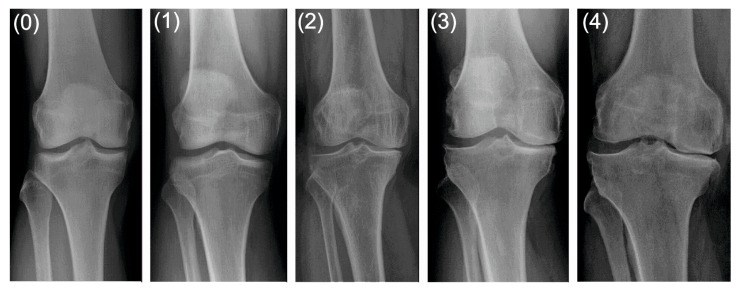
Grades of degenerative changes on the Kellgren-Lawrence scale: 0—none, 1—doubtful, 2—minimal, 3—moderate, 4—severe.

The control group comprised 14 patients with grade 0 and 11 patients with grade 1 degenerative changes on the K–L scale, whereas 39 and 18 patients from the study group were diagnosed with grade 3 and 4 degenerative changes on the K–L scale, respectively [11]. Examples of X-rays are presented in Figure 2.

**Figure 3:**
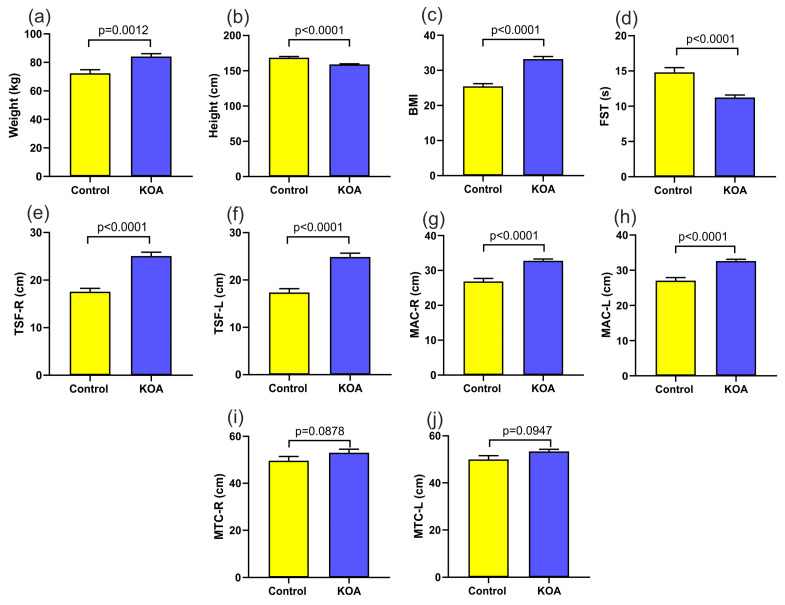
(**a**) body weight in the patient groups; (**b**) height in the patient groups; (**c**) BMI in the patient groups; (**d**) 30 s sit-to-stand test; (**e**) right triceps skin fold; (**f**) left triceps skin fold; (**g**) right arm circumference; (**h**) left arm circumference; (**i**) right thigh circumference; (**j**) left thigh circumference. Control: patients with 0–1 grade on the K–L scale; KOA: patients with 3–4 grade on the K–L scale.

The mean age of the patients was 69 ± 7.5 years (49–84 years) in the study group and 50 ± 4.3 years (42–60 years) in the control group. A highly significant statistical relationship was found between the control and the study group in such parameters as body weight (*p* < 0.012), height (*p* < 0.0001), and BMI (*p* < 0.001). The results of the analysis are presented in Figure 3.

There was a clear statistical difference in the results of the 30 s sit-to-stand test. The mean value was 14.8 repetitions in the control group and 11.2 repetitions in the study group (*p* < 0.0001). A clear statistical difference was found in the thickness of the right (*p* < 0.0001) and left (*p* < 0.0001) arm skin folds between the analyzed groups. Similar relationships were found in the measurements of the right (*p* < 0.0001) and left (*p* < 0.0001) arm circumferences. There was no statistically significant difference in the thigh circumferences between the groups. No statistically significant differences in the size of the tibial tubercle fold were found between the groups. The results of the anthropological examinations, as well as the height, weight, and BMI, are presented in Table 2.

### 3.2. Electrical Bioimpedance Assessment

The following body composition parameters: Total Body Water (TBW), Extracellular Water (ECW), Intracellular Water (ICW), Fat Free Mass (FFM), and Fat-Mass (FM) were assessed using BIA. Detailed results are presented in Table 3.

Clear statistically significant differences were found in the fat mass (FM) between the analyzed groups (*p* = 0.0244 for % FM and *p* = 0.0489 for kg FM). A relationship close to statistical significance was observed in the percentage of Fat-Free Mass and in the percentage of Total Body Water between the groups.

The following electrical parameters were assessed: membrane potential (C membrane), electrical resistance (R—resistance) measured at current frequencies of 5, 50, 100, and 200 kHz, capacitive reactance (reactance—Xc), impedance (Z), and phase angle (phi—Φ). The results are summarized in Table 4.

No statistically significant differences were found between the control and the study group in the following electrical parameters: membrane potential (C membrane) as well as electrical resistance (R—resistance) parameters measured at current frequencies of 5, 50, 100, and 200 kHz, capacitive reactance (reactance—Xc), impedance (Z), and phase angle—(Ph—Φ).

## 4. Discussion

Osteoarthritis is a disease of unknown etiology. Many different factors are regarded as initiators of the cartilage and bone degradation process. One of these factors triggering the development and progression of the disease is excessive body weight [14]. Overweight and obesity increase the risk of many diseases, primarily through the metabolic effects of adipose tissue products, i.e., adipokines, whose immunomodulatory activity contributes to the activation of local and systemic inflammatory processes and exacerbation of the catabolic process in the development of OA [15,16]. Many studies have confirmed increased concentrations of adipose tissue products in KOA [17,18]. Adipose tissue has an impact on the progression of arthrosis through not only metabolic but also mechanical action. Most investigations have shown that subcutaneous fat tissue (SFT) is associated with knee osteoarthritis, and visceral adipose tissue is one of the most important risk factors for the disease [19]. As reported by Collins et al., adipose tissue is a critical antagonist of the health and integrity of hyaline cartilage and a body weight-independent factor of systemic inflammation, synovitis, muscle weakness, and subchondral sclerosis [20].

The aging of societies and the current obesity epidemic Impact and predispose to the development of degenerative joint changes. Considering the current lack of treatment options for degenerative changes, it is worth considering the introduction of diets to inhibit or slow down these changes [21]. With an increase in body weight in the context of metabolic syndrome and an increase in fat tissue mass, there is an acceleration in the development of degenerative changes, especially in the joints of the hands and knees. Introducing an appropriate diet and increasing physical activity can slow down the degenerative process [22,23]. Epidemiological studies indicate that the degree of degenerative changes is associated with serum cholesterol levels. In the Chingford study, a statistically significant moderate increase in serum cholesterol concentration was observed in women with the development of degenerative changes in the knee joints [24]. An increase in cellular cholesterol levels leads to increased cytotoxicity, and an increase in serum levels may cause the activation of pro-inflammatory eicosanoids [25,26]. It has also been demonstrated that cholesterol accumulates in the cartilage, likely due to disturbances in its metabolism with the development of OA [27,28]. Furthermore, it has been observed that LDL (low-density lipoprotein cholesterol) affects the development and progression of degenerative joint disease, especially the oxidized form of LDL, which causes synovial membrane inflammation and cartilage destruction [29]. Therefore, treatment aimed at reducing cholesterol levels in the blood, both through statin use and diet, may have an impact on slowing the progression of degenerative joint disease [30].

During the menopausal period, there are unfavorable changes in body composition, an increase in body weight, and the accumulation of adipose tissue [31]. Additionally, there is a weakening and atrophy of muscle mass, contributing to an increased risk of developing metabolic diseases in older women. Insulin resistance, hyperglycemia, hypertension, hypercholesterolemia, cardiovascular diseases, and central obesity develop, which can lead to the onset of metabolic syndrome (MetS) [32,33]. The reason for the increase in body weight and obesity during the postmenopausal period is the decreased concentration of estrogens in the serum (including estradiol). Additionally, the reduction in estrogen levels influences the dysregulation of the body’s energy balance, although this process has not been fully elucidated to date [34]. Animal studies have shown that estrogens promote the maintenance of a negative energy balance by reducing food intake and increasing energy expenditure. This reduces the risk of weight gain and obesity [35]. The decrease in energy expenditure during menopause is likely due to a combination of a weakened resting metabolism, loss of lean body mass, and reduced physical activity levels. These changes can be associated with aging processes and hormonal changes [36]. In postmenopausal women, there is not only an increase in body weight but also a change in the distribution of adipose tissue [37]. Estrogens favor peripheral fat storage, primarily in the subcutaneous gluteal and femoral regions, while androgens (mainly testosterone) stimulate the accumulation of visceral fat in the abdominal cavity. The accumulation of adipose tissue in the abdominal cavity predisposes to an increased risk of metabolic diseases, to which postmenopausal women are vulnerable [38]. In addition to increased weight gain and changes in adipose tissue distribution, the menopausal period can directly influence changes in fat tissue metabolism by reducing estrogen activity and increasing androgen levels [39].

The BMI is the main parameter used to assess the ratio of body weight to height. In the present study, significantly higher BMI values were determined in the study group. This finding is in agreement with the results reported by Funck-Brentano, who has proved that knee and hip osteoarthritis is a result of excessive loads on the joints, and body weight reduction alleviates these ailments and slows down the disease process [8,40].

However, the BMI parameter does not provide detailed information about the body composition. Electrical bioimpedance analysis facilitates a more accurate assessment of the relationships between various body structures and their impact on the development of degenerative changes. In their study, Lee et al. have found that the progression of osteoarthritis is accompanied by sarcopenia, weakening, and muscle tissue remodeling [41]. The mechanism of changes occurring in the muscle structure has not been elucidated to date. In their study, Noehren et al. have proved that osteoarthritis is accompanied by an increase in the concentration of inflammatory markers leading to muscle fibrosis [42]. Rojas-Rodriguez et al. have found that insulin resistance and systemic inflammation developing in metabolic syndrome (often diagnosed in obese patients) can cause changes in striated muscle tissue, resulting in muscle atrophy and weakness [43]. As hypothesized by Pisters, pain caused by intra-articular damage is responsible for muscle weakness and atrophy [44].

The STS test is used to assess muscle strength and function. The ability to perform the sit-to-stand (STS) movement is important for maintaining physical independence and may be one of the most important functional measures of physical capacity [45]. The 30 s sit-to-stand test is correlated with knee extensor peak torque (strength). The present study showed a clear difference in the performance of the test between the groups. The lower number of repetitions in the study group may indicate lower physical capacity of the patients, weakness and reduced extensor muscle mass, and pain caused by the degenerative changes. Turcot et al. confirmed that, among the clinical factors, this modified movement pattern adopted by those with KOA is related to pain, muscle weakness, and the need to unload the affected lower limb [46].

As shown by Su et al., patients with knee OA performed the STS more slowly than the healthy elderly. Su et al. explained the increase in the time to perform the STS task with the forward lean of the trunk [47].

In their study, Petrella et al. compared patients with unilateral and bilateral degenerative changes in the knee joints. The STS test revealed that patients with bilateral KOA exhibited a lower magnitude of TSM (total support moment) on the more affected limb and greater trunk flexion while straightening the body to achieve an upright position. In addition, the patients with bilateral OA experienced greater pain and were weaker than those in the unilateral OA group. [48]

The Total Body Water (TBW) decreases with aging mainly due to a decrease in Intracellular Water (ICW) [49], which may be a result of muscle remodeling [50]. Muscle tissue consists of 70% water. The ICW content decreases with age, which may reflect a decrease in the hydration of muscle cells and may influence the mechanical and metabolic functions of muscle tissue [51]. In the present study, the study group was characterized by a lower amount of TBW, probably due to the reduction in muscle mass in KOA in relation to fat mass, which confirms the observations mentioned above. Additionally, obese females are characterized by lower TBW per body weight unit and higher ECW per TBW unit in comparison with females with normal weight. These results indicate that the loss of muscle tissue and fat remodeling are accompanied by an increase in the ECW value [52]. This was not confirmed in the present study, as no statistical significance in the ICW and ECW levels was found between the analyzed groups.

In the present study, the FM (fat mass) factor was statistically significantly higher in the patients from the study group. Changes in cartilage and the development of arthrosis are clearly associated with an increase in FM and BMI [53]. This was confirmed by Sowers et al., who reported a clear relationship between body weight and the severity of knee osteoarthritis, and by an observation of particular susceptibility of subjects with a high value of FM to the development of knee arthrosis [54]. In their study, Conroy et al. evidenced the impact of fat mass on the severity of KOA [55]. In contrast, Abbate et al. did not observe such relationships [56]. In turn, Sowers et al. employed the bioimpedance technique for analyses of the body composition and showed an increasing risk of severe radiographic osteoarthritis and joint space narrowing associated with increasing fat and muscle mass (in separate analyses), although a greater variability was explained by the correlation with muscle mass [54].

In the present study, a clear difference in free fat mass was found between the study groups. This indicates a decrease in muscle mass in the group of KOA patients, as in a study conducted by Davidson et al., who reported fatty infiltration in thigh and calf muscles accompanying degenerative changes in the knee joint [57]. Visser et al. determined the relationship between adipose tissue mass and skeletal muscle mass in knee osteoarthritis. The researchers found that weight reduction with simultaneous improvement of muscle strength and mass may be useful for the prevention and treatment of knee osteoarthritis [58]. Similarly, other authors have shown that fat mass reduction minimizes the risk of knee osteoarthritis and reduces the severity of its symptoms [59,60].

The weight loss process is accompanied by loss of fat mass and reductions in muscle mass and strength. Therefore, it is highly important to introduce physical exercise simultaneously with a diet, as it can improve muscle strength, increase muscle mass, and accelerate metabolism. As confirmed by Henriksen et al., weight loss improves physical fitness but leads to loss of leg muscle tissue and knee muscle strength, which confirms the necessity to restore or increase muscle mass during the weight loss period [61].

However, a substantial weight loss in the elderly may intensify the development of osteoporotic changes and increase the risk of such complications as femur fractures [62]; therefore, although excess body weight increases the mechanical load on the lower extremities, it may be a protective factor in this age group. Weight loss as a result of intensive dietary intervention, with or without exercise, leads to bone loss in the hip joint and femoral neck in overweight and obese elderly adults with osteoarthritis [63].

The present study showed a clear difference in the arm circumference and skin fold thickness in favor of the study group, which may indicate that the patients can be classified as metabolically healthy obese individuals [64]. Aging is often associated with an uneven distribution of body fat, which results in increased central adiposity with slender arms and legs (sarcopenic obesity) caused by muscle wasting in middle-aged and elderly people, contributing to poor metabolic health [65]. Skin fold measurements are not normally used in the assessment of obesity in patients with degenerative changes, but they may be part of the assessment of the effectiveness of diet or physical exercise aimed at body weight reduction.

Study limitations include a relatively small sample size in the control and study groups. However, we implemented rigorous recruitment criteria to ensure the homogeneity of the groups. Furthermore, the lack of assessment of specific fat tissue markers in synovial fluid and serum may impede a comprehensive understanding of the adipose tissue–osteoarthritis relationship. Further research should focus on the exploration of the metabolic activity of adipose tissue, the possibility of activation of inhibitors of the action of adipokines and the related inflammatory process, and the introduction of dietary and rehabilitation programs to reduce FM and increase FFM in order to inhibit the development of KOA.

## 5. Conclusions

The risks of KOA development and progression are closely related to an increase in adipose tissue mass in low-grade inflammation mechanisms [66] and biomechanical factors [67,68]. The introduction of a diet and physical activity results in weight loss, and even a 1% weight loss is largely associated with retardation of tibial plateau cartilage loss and the progression of KOA [69], which may influence cartilage health and mitigate osteoarthritis symptoms.

Based on the present results, it can be concluded that the relationship of overweight and obesity with KOA in postmenopausal females is associated mainly with the level of adipose tissue with its metabolic activity and its consequences. In clinical practice, reductions in FM with a simultaneous increase in FFM in obese postmenopausal females may slow down the degenerative process in joints.

## Figures and Tables

**Table 1:** The Kellgren and Lawrence classification of the severity of osteoarthritis.

grade 0 (none)	definite absence of X-ray changes of osteoarthritis
grade 1 (doubtful)	doubtful joint space narrowing and possible osteophytic lipping
grade 2 (minimal)	definite osteophytes and possible joint space narrowing
grade 3 (moderate)	moderate multiple osteophytes, definite narrowing of joint space and some sclerosis, and possible deformity of bone ends
grade 4 (severe)	large osteophytes, marked narrowing of joint space, severe sclerosis and definite deformity of bone ends.

**Table 2:** Anthropometric parameters and results of the functional tests of the examined patients.

Variable	Control *n* = 25				KOA *n* = 58			
	Mean	Minimum	Maximum	SD	Mean	Minimum	Maximum	SD
age (years)	50.8000	42.0000	60.0000	4.28174	68.7241	49.0000	84.0000	7.63370
body weight (kg)	72.4000	52.0000	98.0000	12.52331	84.1379	53.0000	128.0000	15.39019
height (cm)	168.5200	150.0000	182.0000	8.87938	159.1034	147.0000	172.0000	5.86901
BMI	25.4564	20.2000	34.6400	3.82058	33.2153	21.2306	47.5907	5.70809
30 s sit-to-stand test	14.8000	7.0000	20.0000	3.41565	11.2414	4.0000	16.0000	2.60438
left triceps skin fold	17.3600	11.0000	26.0000	3.99875	24.3448	10.0000	40.0000	6.45239
right triceps skin fold	17.5600	11.0000	24.0000	3.55996	24.5862	10.0000	38.0000	6.44010
left arm circumference	27.0400	21.0000	39.0000	4.42982	32.6207	23.0000	42.0000	3.88803
right arm circumference	26.8400	19.0000	38.0000	4.42229	32.7931	24.0000	43.0000	3.79639
left thigh circumference	49.9600	29.0000	60.0000	8.01810	52.6379	12.0000	89.0000	10.84289
right thigh circumference	49.6400	29.0000	65.0000	8.97626	53.0345	12.0000	89.0000	11.26704

Control: patients with 0–1 grade on the K–L scale; KOA: patients with 3–4 grade on the K–L scale.

**Table 3:** Parameters of the body composition in the analyzed groups.

Variable	Control *n* = 25				KOA *n* = 58				
	Mean	Minimum	Maximum	SD	Mean	Minimum	Maximum	SD	*p*
TBW (L)	36.93346	26.19136	49.28473	5.67032	38.10232	26.19136	58.31367	6.16898	0.4199
TBW (%)	48.53787	36.55865	58.88215	5.97969	46.02176	34.35431	60.17147	5.74760	0.0743
ECW (L)	16.24664	11.05576	22.24771	2.54051	16.70618	11.05576	24.68876	2.47154	0.5555
ECW (%)	44.05256	36.26720	51.13032	3.16313	44.05635	35.75701	52.13185	3.40034	0.9962
ICW (L)	20.68682	15.13559	30.34301	3.57742	21.39613	14.60117	33.62491	4.15509	0.4646
ICW (%)	55.94744	48.86968	63.73280	3.16313	55.94365	47.86815	64.24300	3.40034	0.9962
FM (kg)	26.77406	13.91347	46.55254	10.15562	31.70627	12.45897	58.90535	10.37543	0.0489 *
FM (%)	33.69143	19.55990	50.05649	8.16898	37.12874	17.79853	53.06788	7.85191	0.0244 *
FFM (kg)	50.57554	35.78054	67.32887	7.89157	52.05235	35.78054	79.66349	8.42756	0.4457
FFM (%)	66.30857	49.94351	80.44010	8.16898	62.87126	46.93212	82.20147	7.85191	0.0746

Total Body Water (TBW), Extracellular Water (ECW), Intracellular Water (ICW), Fat-Free Mass (FFM), and Fat Mass (FM). Control: patients with 0–1 grade on the K–L scale; KOA: patients with 3–4 grade on the K–L scale. * *p*–values below 0.05 indicate a statistically significant difference.

**Table 4:** Values of BIA measurements in the analyzed groups—electrical parameters.

Variable	Control *n* = 25				KOA *n* = 58				
	Mean	Minimum	Maximum	SD	Mean	Minimum	Maximum	SD	*p*
C membrane	1.854	1.2365	4.111	0.6831	1.905	0.9029	6.085	0.9253	0.8061
Z (5)	542.568	410.3825	682.012	76.0506	551.540	388.2021	682.012	70.8498	0.6060
phi (5)	2.691	1.6245	4.932	0.8588	2.777	1.6245	5.228	0.7596	0.6494
R5	541.989	410.1253	681.171	76.2156	550.831	387.8147	681.171	70.6674	0.6109
Xc5	26.013	13.0737	56.190	10.8260	26.939	13.0737	56.190	8.8286	0.6838
Z (50)	480.946	371.0419	609,505	61.1055	486.395	344.3167	609.505	60.4426	0.7082
phi (50)	4.823	3.5952	7.724	0.9767	4.984	3.4007	7.944	0.9734	0.4895
R50	479.527	354.8673	607.329	60.8928	484.494	342.8439	607.329	60.2713	0.7322
Xc50	40.580	28.4855	69.314	10.3761	42.175	25.2161	69.314	9.3318	0.4918
Z (100)	459.260	354.7543	583.725	57.6525	463.829	327.5560	583.725	57.7581	0.7416
phi (100)	4.464	2.8827	7.171	0.8603	4.448	2.3019	8.191	1.0310	0.9455
R100	457.813	353.7130	581.857	57.5310	462.358	326.3990	581.857	57.6929	0.7425
Xc100 ohms	35.673	21.2281	60.105	7.9104	35.813	16.4499	60.105	8.7250	0.9452
Z (200)	441.147	341.2459	562.079	55.9852	445,622	314.2859	562.079	56.2306	0.7399
phi (200)	2.718	0.1151	6.075	1.3409	2.558	0.1093	8.743	1.6813	0.6752
R200 ohms	440.537	340.8191	560.821	55.9809	444.975	313.6346	560.821	56.2907	0.7421
Xc200	20.656	0.8208	48.060	10.5455	19.578	0.8208	56.785	12.7551	0.7117
Z c	488.040	369.9541	616.375	67.5612	495.728	348.5110	616.375	63.7676	0.6219

Membrane potential (C membrane), electrical resistance (R—resistance) measured at current frequencies of 5, 50, 100, and 200 kHz, capacitive reactance (reactance—Xc), impedance (Z), and phase angle (phi—Φ). Control: patients with 0–1 grade on the K–L scale; KOA: patients with 3–4 grade on the K–L scale.

## Data Availability

The data presented in this study are available upon request from the corresponding author.
